# Associations between Dietary Patterns and Malnutrition, Low Muscle Mass and Sarcopenia in Adults with Cancer: A Scoping Review

**DOI:** 10.3390/ijerph19031769

**Published:** 2022-02-04

**Authors:** Annie R. Curtis, Katherine M. Livingstone, Robin M. Daly, Laura E. Marchese, Nicole Kiss

**Affiliations:** 1 Institute for Physical Activity and Nutrition Research, School of Exercise and Nutrition Sciences, Deakin University, Geelong, VIC 3220, Australia; k.livingstone@deakin.edu.au (K.M.L.); robin.daly@deakin.edu.au (R.M.D.); lmarchese@deakin.edu.au (L.E.M.); nicole.kiss@deakin.edu.au (N.K.); 2 Allied Health Department, Peter MacCallum Cancer Centre, Melbourne, VIC 3000, Australia

**Keywords:** dietary patterns, cancer, malnutrition, muscle mass, sarcopenia

## Abstract

Dietary patterns examine the combinations, types and quantities of foods consumed in the diet. Compared to individual nutrients, dietary patterns may be better associated with cancer-related malnutrition, low muscle mass and sarcopenia. This scoping review identified associations between dietary patterns, assessed using data-driven methods (i.e., statistical methods used to derive existing dietary patterns) and hypothesis-orientated methods (i.e., adherence to diet quality indices), and malnutrition, low muscle (lean) mass and sarcopenia. MEDLINE, Embase and CINAHL databases were searched up to September 2021. Of the 3341 studies identified, seven studies were eligible for review. Study designs included experimental (*n* = 5) and observational (*n* = 2), and people with prostate, ovarian and endometrial, bladder, breast, and gastrointestinal cancers. One study used data-driven methods to derive dietary patterns, finding adherence to a ‘fat and fish’ diet was associated with lower odds of low muscle mass. Two studies examined adherence to hypothesis-orientated methods including the Mediterranean Diet Adherence Screener and Healthy Eating Index 2010 and four studies used ‘non-traditional’ approaches to analyse dietary patterns. Hypothesis-orientated dietary patterns, developed to improve general health and prevent chronic disease, and ‘non-traditional’ dietary patterns demonstrated inconsistent effects on muscle (lean) mass. All studies investigated muscle (lean) mass, omitting malnutrition and sarcopenia as cancer-related outcomes. This scoping review highlights the limited research examining the effect of dietary patterns on cancer-related outcomes.

## 1. Introduction

Malnutrition and muscle loss are some of the well-recognised consequences of cancer. Cancer type, disease stage and treatment modality can all impact upon nutritional intake and status of people with cancer, leading to malnutrition and muscle loss [1]. Malnutrition is a condition resulting from compromised intake or uptake of foods and nutrients and may be associated with disease-related inflammation [2]. Cancer-related malnutrition affects 25% to 60% of people with cancer with differences relating to the nutritional assessment method used, cancer diagnosis or treatment modality [3,4,5]. Low muscle mass is a key characteristic of malnutrition and is one of the three phenotypic criteria of the current Global Leadership Initiative on Malnutrition (GLIM) diagnosis of malnutrition [6]. It is estimated that low muscle mass impacts up to 79% of people with certain cancer diagnoses [7]. Malnutrition and/or low muscle mass are associated with poorer cancer-related outcomes, including increased risk of treatment toxicities and poorer treatment outcomes [8], worse quality of life [9,10] and increased mortality [11,12] compared to those who are well-nourished or have a normal muscle mass. Sarcopenia, encompassing low muscle mass plus low muscle strength and/or poor physical performance [13,14], is also associated with post-operative complications [15,16]. To date, few oncological studies have examined the effects of sarcopenia, instead using the term ‘sarcopenia’ to define the presence of low muscle (lean) mass alone. 

Given a current lack of effective pharmacological solutions for low muscle mass and sarcopenia [17], nutritional interventions are essential to patient care. The potential benefits of certain nutrients in attenuating and managing muscle loss in cancer have been summarised previously, including protein and amino acids, omega-3 fatty acids, carnitine, creatine and certain vitamins and minerals [18]. Current international nutrition guidelines by the European Society for Clinical Nutrition and Metabolism (ESPEN) recommend a protein intake above 1 g/kg/day and up to 1.5 g/kg/day to reduce lean mass deterioration from cancer and anti-cancer treatments [19]. Additionally, these guidelines specify that vitamins and minerals are recommended in amounts that are adequate to meet daily allowances, and for people with advanced cancer, who are undergoing chemotherapy, supplementation with long-chain omega-3 fatty acids is recommended to improve appetite and food intake [19]. However, dietary intake is complex, with interrelationships between foods and beverages, nutrients and other dietary components. Therefore, dietary analyses and subsequent dietary recommendations should account for this multiplicity.

Dietary patterns consider the complex nature of dietary intake and encompass nutrients, foods and beverages, and eating occasions [20]. According to the United States Department of Agriculture (USDA), dietary patterns are “the quantities, proportions, variety, or combination of different foods, drinks, and nutrients in diets and the frequency with which they are habitually consumed” [21]. Dietary patterns are assessed using two main approaches: (i) hypothesis-orientated or diet quality methods, which assess dietary intake based on adherence to a priori nutritional indexes, dietary guidelines or recommendations designed to prevent chronic disease and improve general health; and (ii) data-driven methods, which include a range of statistical approaches to identify existing dietary patterns from dietary data [20]. Then, hybrid methods combine the two previous approaches to derive existing dietary patterns, using a priori knowledge of diet-disease related variables, such as nutrient intakes, biomarkers, or risk factors of disease. 

Dietary patterns are emerging as an important factor in cancer research. Systematic reviews and meta-analyses of observational studies in various cancer types have examined the associations between dietary patterns and cancer risk, reporting approximately 10–25% decreased odds of developing cancer with higher adherence to ‘prudent’ or ‘healthy’ dietary patterns derived using data-driven methods [22,23,24]. A reduction in cancer risk is also observed with adherence to a priori dietary indexes such as the Mediterranean Diet Score (up to 58% lower risk) and Healthy Eating Index (up to 56% lower risk), and poorer adherence to the Dietary Inflammatory Index (up to 200% increased risk with a more inflammatory diet) [25,26]. Reviews and meta-analyses of observational and interventional studies have further examined the impact of dietary patterns on cancer-related outcomes in cancer survivors with ‘healthy’, high quality ‘prudent’, and Mediterranean diets, but not plant-based diets, associated with reduced risk of mortality (24% reduced risk of all-cause mortality in breast cancer with ‘healthy dietary pattern’) and cancer recurrence [27,28,29]. However, evidence for the role of dietary patterns in optimising outcomes during cancer treatment, including the prevention and management of malnutrition and low muscle (lean) mass, is only now beginning to be explored. Dietary patterns allow for investigation of the whole diet, reflecting real-world intake, and as such dietary patterns may be effective at preventing and managing malnutrition, low muscle (lean) mass and sarcopenia in people with cancer. Therefore, the aim of this scoping review was to synthesise the literature assessing associations between dietary patterns and malnutrition, low muscle (lean) mass and sarcopenia in adults with cancer and to highlight gaps in existing knowledge.

## 2. Materials and Methods

Scoping reviews are used to determine the scope of emerging evidence and examine the volume and focus of literature on a given topic [30]. As such, the theoretical uses of a scoping review met our aims in a research field which was theorized by authors to be limited in quantity and would be heterogeneous in terms of dietary patterns approaches examined. This scoping review was conducted and reported in accordance with the Preferred Reporting Items for Systematic Reviews and Meta-analysis—Extension for Scoping Reviews (PRISMA-ScR) [31].

### 2.1. Eligibility Criteria 

Studies included in this scoping review were peer-reviewed original research studies, published in English from inception to the date of the final literature search (10 September 2021) and available in full text format. Eligible studies examined associations between dietary patterns and malnutrition and/or low muscle mass and/or sarcopenia in adults with any cancer diagnosis. Dietary patterns, including those assessed using data-driven, hypothesis-oriented or hybrid approaches, were the primary exposures of interest. Due to the anticipated limited quantity of literature using traditional dietary pattern approaches, studies were included if they examined ‘non-traditional’ dietary patterns approaches (i.e., adherence was not assessed by an overall dietary pattern score), provided the authors adequately described both the foods consumed and the methods used to assess adherence to the dietary pattern. Outcomes of interest were: (i) presence of malnutrition, assessed using validated measures (i.e., not use of albumin biomarkers); (ii) objectively assessed low muscle mass and; (iii) objectively assessed sarcopenia, including the phenotypic components of muscle strength and physical performance. Excluded were studies which did not meet the aims of this scoping review and reported on individual nutrients, foods, or supplements.

### 2.2. Search Strategy 

#### 2.2.1. Information Sources

A systematic literature search was conducted in MEDLINE Complete, Embase and CINAHL Complete databases on 10 September 2021. For completeness, the reference lists of included studies were hand-searched by one author (A.R.C.) to identify studies not discovered during the original literature search. The search strategy was developed and refined through discussion between authors and in collaboration with an experienced health science librarian to ensure an appropriate and robust search of the literature. 

#### 2.2.2. Literature Search 

The literature search was confined to study titles and abstracts. Key search terms included ‘cancer’, ‘diet’, ‘malnutrition’, ‘muscle mass’, ‘sarcopenia’, ‘muscle strength’ and ‘physical function’ and synonyms of the same. Medical subject headings were additionally searched for each term. The MEDLINE Complete search strategy, including medical subject headings and applied limiters is available in Table A1 (Appendix A).

#### 2.2.3. Selection of Sources of Evidence 

Two authors (A.R.C. and L.E.M.) independently screened studies against the eligibility criteria in two stages. Firstly, the title and abstract of identified studies were scrutinized; any disagreements in eligibility were resolved through discussion by the same authors. Secondly, full text versions of relevant studies were sourced and screened independently against the eligibility criteria. When required, consensus was reached through discussion between authors and in the case of disagreement or uncertainty a third and fourth author (K.M.L. and N.K.) were consulted. 

### 2.3. Data Charting Process 

A data extraction chart was developed by one author (A.R.C.) and adapted using feedback from two other authors (K.M.L. and N.K.). Data charting was conducted for eligible studies in Microsoft Excel by one author (A.R.C.) and verified for accuracy and clarity by two authors (L.E.M. and N.K.). Information regarding study design and setting, population (number of participants, age, sex, and cancer diagnosis), exposure variables (dietary intake assessment method and dietary patterns), outcome variables (malnutrition, muscle (lean) mass and sarcopenia), time of assessments and results were summarised and evaluated. 

### 2.4. Synthesis of Results 

A narrative synthesis was conducted to summarise the major findings from the included studies. Results were presented by grouping individual studies based on their dietary patterns approach, including data-driven, hypothesis-orientated, and dietary patterns assessed using ‘non-traditional’ approaches, an approach used in previous reviews of dietary patterns research to present results [32]. This approach allowed each study to be adequately presented and summarised, knowledge gaps to be highlighted, and opportunities for future research to be identified [31]. 

## 3. Results

**Figure 1:**
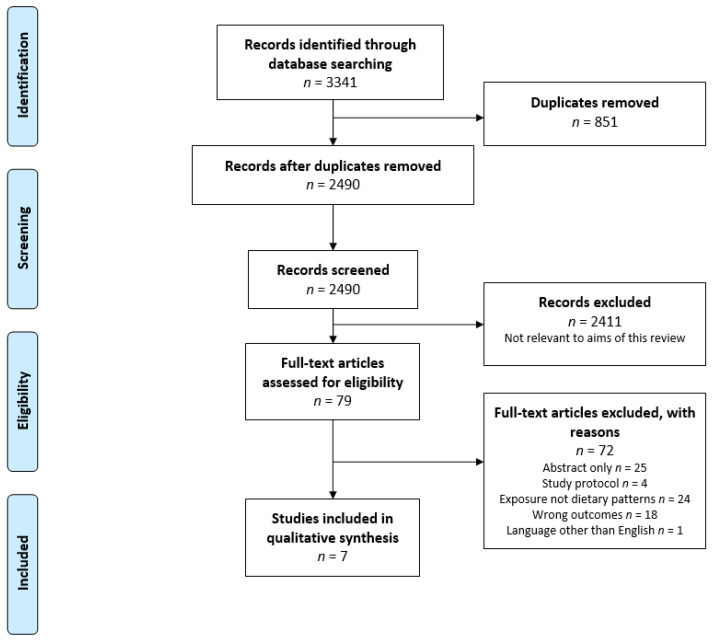
PRISMA flow diagram of search results from MEDLINE, Embase and CINAHL databases.

In total 3341 potentially relevant studies were identified from the three selected databases. Of these, 851 were duplicates, yielding 2490 studies to be screened. After review of titles and abstracts, 2411 studies did not meet the eligibility criteria and were excluded. Full text versions of the remaining 79 studies were screened, and a further 72 studies were excluded. Seven eligible studies were identified for data charting and review. Figure 1 presents the PRISMA flow diagram, outlining the study selection process. 

### 3.1. Study Characteristics

The seven included studies were published between 2018 and 2021. Study designs included two randomised controlled trials [33,34], three non-randomised controlled trials [35,36,37] and two observational cross-sectional studies [38,39]. The number of participants ranged from 23 to 285, including adults aged between 52 to 74 years. People with prostate, ovarian, endometrial, bladder, breast, and upper and lower gastrointestinal cancers were included. One study included treatment naive participants [38], two studies included people undergoing radiotherapy [35,37], one study included people during rehabilitation [36] and three studies included people who were diagnosed or treated between 2 to over 5 years prior to assessment [33,34,39].

### 3.2. Nutrition and Muscle-Related Outcomes Measured

All included studies reported on measures of muscle mass, lean mass or muscle cross-sectional area. Bioelectrical impedance analysis (BIA) was used for estimation of changes in fat-free mass (kg) [35,37] and dual energy X-ray absorptiometry (DXA) was used for estimation of changes in total body lean mass (kg) [33,34]. Changes in skeletal muscle mass (kg) were also estimated using measures derived from both BIA and DXA [35,36,37]. Computed tomography (CT) was used to derive a skeletal muscle index (SMI; skeletal muscle area [cm^2^] divided by height squared [m^2^]) at the third lumbar vertebrae (L3) [38,39]. Where CT were used, Hounsfield Unit (HU) thresholds for segmentation of skeletal muscle tissue cross-sectional area ranged from −29 to 150 HU with low muscle mass defined according to sex-specific SMI cut-offs of ≤52.4 cm^2^/m^2^ for men and ≤38.5 cm^2^/m^2^ for women [39] or <43 cm^2^/m^2^ for men if BMI < 25 kg/m^2^ or <53 cm^2^/m^2^ if BMI > 25 kg/m^2^ and <41 cm^2^/m^2^ for women [38]. No studies assessed malnutrition or sarcopenia as outcomes.

### 3.3. Dietary Intake Assessments

Dietary intake was assessed via food frequency questionnaire (FFQ; Portuguese-specific semi-quantitative questionnaire and Diet History Questionnaire II) [38,39], food diaries [34,35,36,37] or one-month diet history (Wollongong Dietary Inventory) [33].

### 3.4. Dietary Patterns Methods

Dietary pattern approaches were heterogeneous across the seven studies. One study used data-driven methods, where principal component analysis (PCA) was conducted [38]. Two studies used hypothesis-orientated methods to assess diet-quality, namely the Mediterranean Diet Adherence Screener and Healthy Eating Index 2010 [33,39]. Four studies examined ‘non-traditional’ dietary patterns, namely measuring adherence to the ketogenic diet and ‘low carb’ diets via food diaries and biological samples [34,35,36,37]. Characteristics of the seven studies, including key findings, are reported in Table 1.

### 3.5. Data-Driven Dietary Patterns

One cross-sectional study used PCA to derive dietary patterns and determine their association with the odds of having low muscle mass (derived from SMI) [38]. Four major dietary patterns were identified from semi-quantitative FFQ including: (i) high-fat dairy products, fried snacks, and processed meat diet; (ii) legumes, vegetables and fruit diet; (iii) fat and fish diet; and (iv) alcohol, cereal and animal protein diet. Overall, 32% of participants had low muscle mass. The participants in the second and third tertiles of adherence to the ‘fat and fish’ diet (summarised in Table 2) had significantly lower odds of having low muscle mass compared with participants in the first tertile. No significant associations were observed for the remaining three dietary patterns.

### 3.6. Hypothesis-Orientated Dietary Patterns

Two studies used dietary indexes to assess associations between diet quality and lean- or muscle mass [33,39]. The first was a 12-week randomised controlled trial, which used the Mediterranean Diet Adherence Screener (summarised in Table 2) to assess adherence to the Mediterranean dietary pattern and its association with lean mass [33]. Dietary data were collected using the Wollongong Dietary Inventory, a one-month diet history [40]. Participants in the Mediterranean diet group demonstrated high adherence to the Mediterranean dietary pattern, with 81% of participants achieving a score of ≥75% on the Mediterranean Diet Adherence Screener by the study’s conclusion. The Mediterranean diet was associated with significantly lower lean mass at 8-weeks and non-significantly lower lean mass at 12-weeks, compared to the usual care group. The between-group differences were due to a non-significant reduction in lean mass between baseline and weeks 8 and 12 for the Mediterranean diet group, while lean mass remained stable in the usual care group.

The second study used the Healthy Eating Index 2010 (summarised in Table 2) to assess cross-sectional associations between diet quality using the Diet History Questionnaire II, a food frequency questionnaire, and muscle mass (derived from SMI) [39]. Overall, 72% of men and 55% of women had low muscle mass. Multivariate linear regression analysis (adjusted for age, gender, and race) demonstrated that diet quality was not associated with muscle mass.

### 3.7. Dietary Patterns Assessed Using ‘Non-Traditional’ Approaches

Four studies used ‘non-traditional’ approaches to analyse dietary patterns. All four studies investigated variations on the ketogenic dietary pattern and in one study a ‘low carb’ diet was also assessed (ketogenic and ‘low carb’ dietary patterns are summarised in Table 2). Associations between dietary patterns and lean-, fat free- and skeletal muscle mass were investigated [34,35,36,37]. Findings from these studies were unfavourable overall. One 12-week randomised controlled trial investigated associations between the ketogenic dietary pattern and lean mass, compared to the lower fat American Cancer Society diet [34]. Approximately 80% of participants adhered to their assigned diet based on the examination of food records and achieving urinary ketone concentrations of approximately 0.5 mmol/L for the ketogenic diet group. The ketogenic diet group appeared to lose lean mass (−0.9 kg) whilst the American Cancer Society diet group maintained lean mass (−0.1 kg). However, after adjusting for baseline conditions, there were no significant differences in lean mass between the ketogenic diet group and American Cancer Society diet group at 12 weeks.

Two non-randomised controlled trials assessed associations between the ketogenic dietary pattern and fat free- and skeletal muscle mass, compared to an unspecified standard diet [35,37]. In both studies, interventions lasted approximately one month (between 34 to 37 days). Adherence to the ketogenic dietary patterns were assessed based on analysis of food diaries and urinary or capillary ketone concentrations. In the first study by Klement et al. 2020 [37], mean (0.72 mmol/L) and median (0.49 mmol/L) fasting ketone concentrations were significantly higher in the ketogenic diet group compared to the standard diet group (mean 0.13, median 0.06 mmol/L; *p* < 2.2 × 10^−16^). Three participants were excluded due to noncompliance with the ketogenic diet. In the second study by Klement et al. 2021 [35] median capillary ketone concentrations in the ketogenic group were 0.6 mmol/L and all participants achieved at least one ketone measurement < 0.4 mmol/L, thus it was reported that all participants tried to comply with the ketogenic dietary pattern. The ketogenic dietary pattern was associated with significant reductions in fat free mass (−1.23 kg) [37] and skeletal muscle mass (−0.71 kg and −0.80 kg) [35,37], whilst a standard diet was associated with small non-significant changes.

Finally, one 20-week non-randomised controlled trial compared a healthy standard diet, ‘low carb’ and ketogenic diet (as part of a multimodal rehabilitation program) for the associations with skeletal muscle mass [36]. Adherence to the respective dietary patterns were assessed by analysis of food diaries plus the analysis of urinary ketones for the ketogenic diet group. Patients in the ketogenic diet group achieved the intended ketogenic ratio (grams of fat divided by grams of carbohydrates plus protein) of 1:6:1, which was significantly higher than the low carbohydrate diet and standard diet groups. The low carbohydrate diet was associated with significant losses of skeletal muscle mass (−0.9 kg), whilst the ketogenic dietary pattern (−0.9 kg) and standard diet (−0.3 kg) were associated with non-significant losses of skeletal muscle mass.

## 4. Discussion

The main finding from this scoping review was that adherence to a ‘fat and fish’ diet, derived using a data-driven dietary patterns approach, may be associated with lower odds of having low muscle mass in people with gastrointestinal cancers. Hypothesis-orientated dietary patterns, developed to improve health and prevent chronic diseases, and adherence to ketogenic or ‘low carb’ dietary pattern were either not associated with or negatively associated with muscle (lean) mass in various cancers. Of the seven studies reviewed, all examined lean-, skeletal muscle- or fat-free mass, omitting malnutrition, sarcopenia as potentially important cancer-related outcomes. Overall, the methods used to analyse dietary patterns varied across studies as did the dietary intake assessment methods (i.e., food frequency questionnaires and food diaries). The small number of eligible studies and their heterogeneity in study design limited our ability to draw definitive conclusions regarding the associations between dietary patterns and cancer-related muscle mass.

### 4.1. Data-Driven Dietary Patterns and Muscle Mass

Only one study used data-driven methods to derive dietary patterns in Portuguese adults with gastrointestinal cancers [38]. This was the only dietary pattern to have a positive effect on muscle mass. High adherence to a ‘fat and fish’ diet, characterised by intake of high fat foods and fish, was associated with approximately 70% lower odds of having low muscle mass [38]. Interestingly, this finding does not align with recent studies of comparable ‘unhealthy’ or ‘high-fat’ diets in healthy adults. Specifically, in a three year prospective study including 757 community dwelling older adults, Granic et al. [43], demonstrated a ‘Traditional British’ diet, encompassing high fat and energy intake from butter, red meat, gravy, potatoes, sweet and desserts was associated with 75% and 150% increased odds of sarcopenia (defined as low SMI and low gait speed or grip strength) at baseline and three years, respectively, compared to those consuming a ‘low butter’ reference diet. Alternatively, in a 15-year longitudinal study including 522 healthy Australian men, Davis et al. [44] found that scoring higher for a ‘Traditional’ dietary pattern, characterised by a greater consumption of fruit and vegetables, nuts, unprocessed fish and red and white meats, predicted a greater SMI (beta-co-efficient: 0.12 kg/m^2^; *p* < 0.05) over the 15 years follow up.

The variation in findings may be due to the differing nutritional needs of people with cancer compared to healthy adults. Nutritional recommendations for people with cancer are commonly enhanced in order to counter the metabolic demands of anticancer treatments and the disease itself [19]. The ‘fat and fish’ dietary pattern derived by Velho et al. [38] was energy dense and contained fish, which is both a source of protein and omega-3 fatty acids, consistent with nutrient-specific recommendations made in international nutritional guidelines for cancer [19]. The ‘fat and fish’ dietary pattern also reflects the principals of a ‘high-energy high-protein’ diet, which is commonly prescribed in clinical practice by nutrition professionals. For example, energy dense ‘discretionary’ foods may be recommended to patients with low appetite to provide sufficient energy and protein in smaller, easy to consume foods. In all, the ‘fat and fish’ dietary pattern may provide adequate energy- and nutrient-dense foods, sufficient to attenuate muscle loss in people with cancer. However, it remains unclear whether a dietary pattern containing ‘healthier’ and less-processed food sources of energy, protein, and omega-3 fatty acids, may be more beneficial and equally palatable to people with cancer, especially those experiencing low appetite or other intake limiting side effects, compared to high saturated fat snacks such as chocolates and cookies.

### 4.2. Hypothesis-Orientated and ‘Non-Traditional’ Dietary Patterns and Muscle Mass

The majority of studies identified in this scoping review used hypothesis-orientated or ‘non-traditional’ methods to assess dietary patterns in association with muscle (lean) mass. Two different hypothesis-orientated dietary indexes were used, the Mediterranean Diet Adherence Screener [33] and Healthy Eating Index 2010 [39]. Biological samples were primarily used to assess adherence to variations of the ketogenic diet [34,35,36,37]. Although the latter are not considered ‘true’ dietary patterns, as they were not assessed using known assessment methods, they provide context to the current body of literature and will be discussed here. All studies found that hypothesis-orientated dietary patterns and ‘non-traditional’ dietary patterns (ketogenic and low carbohydrate diets) were either not associated with muscle (lean) mass [34,39] or were detrimental regardless of cancer type [33,35,36,37].

There are likely multiple reasons why these dietary patterns were not beneficial. Firstly, the hypothesis-orientated dietary patterns described in this scoping review were primarily developed to improve general health and prevent chronic disease, rather than to improve muscle (lean) mass. To date, there are no muscle-specific dietary indexes developed for the purpose of attenuating muscle loss or improving muscle mass, strength, or function in people with cancer. As such, it is unclear whether the dietary indexes examined in this review promote intake of sufficient energy and nutrient profile to meet the increased needs of people with cancer. For example, a review by Romagnolo et al. [45] suggests that despite the positive anti-inflammatory properties of the Mediterranean diet, foods and nutrients which constitute the diet may be insufficient to meet the protein and vitamin D recommendations of people with cancer. Similarly, a ketogenic diet, which is defined by a minimal intake of carbohydrates and high intake of fats, is commonly associated with weight loss. Whilst the majority of studies in this scoping review did not report on energy intake of participants, losses of body mass and fat mass [34,35,36,37] may suggest that energy intake was inadequate to meet the needs of people with cancer, potentially promoting loss of muscle mass.

Another explanation for the negative findings associated with hypothesis-orientated dietary patterns may be related to the foods and beverages which constitute these diets. Each dietary pattern including Mediterranean, Healthy Eating Index and ketogenic and ‘low carb’ diets, are characterised by certain foods. For example, the Mediterranean diet promotes intake of extra-virgin olive oil, fruits and vegetables, wholegrain cereals, nuts and legumes, and a moderate to small amount of fish, meat and dairy products [46]. Therefore, the appropriateness of these dietary patterns for people with cancer must be considered. Nutrition impact symptoms are common among people undergoing treatment for cancer and into survivorship, those such as low appetite, pain, change in dentition or taste function may prevent adequate dietary intake [47]. As such, abundance of low energy, fibrous foods, may not be optimal to meet the nutritional demands of people with cancer, nor be appropriate or palatable for those experiencing nutrition impact symptoms. Nutrition impact symptoms were not reported in the studies in this review; however, their impact on compliance must be considered in this context.

Like data-driven dietary patterns, there is a paucity of studies regarding the role of hypothesis-orientated dietary patterns on muscle (lean) mass in people with cancer. The majority of studies identified in this review included people with prostate [33] ovarian and endometrial [34] and breast cancers [36,37], which are not generally associated with heightened nutritional risk. Therefore, it is difficult to generalise these findings to other cancer types more typically associated with a higher risk of malnutrition and muscle loss, such as lung or gastrointestinal cancers. Baguley et al. [33] examined the effects of a 12-week Mediterranean diet intervention compared to usual care, on lean mass in men with prostate cancer receiving androgen deprivation therapy. Whilst men with prostate cancer are considered at low risk of malnutrition, androgen deprivation therapy is commonly associated with marked changes in body composition, namely loss of muscle mass and gain of fat mass [48]. Therefore, loss of lean mass in this instance may, at least in part, be a product of treatment effects rather than dietary intake.

### 4.3. Implications for Future Research

This scoping review revealed a paucity of studies examining the associations between dietary patterns and low muscle mass in adults with cancer. Of particular note, examination of malnutrition and sarcopenia were entirely absent from the identified literature. Therefore, it remains unclear whether dietary patterns have any impact upon these prevalent conditions. Most included studies employed ‘non-traditional’ approaches to analyse dietary patterns, using biological ketone analysis and food records, to assess adherence to nonhomogeneous ketogenic diets, rather than recognised dietary indexes. Whilst some limited research supports the role of ketogenic diets as a concurrent therapeutic option for cancer, for example, in cases of malignant glioma [49,50], this review demonstrates they have a detrimental effect on muscle mass. A dietary index, reflecting the current knowledge of diet-muscle relationships, rather than to improve general health and prevent chronic disease, may provide further insights into the role of diet on muscle mass. As one does not currently exist, its development should consider the unique nutritional needs and types of foods that are tolerated by people with cancer.

Dietary patterns derived from data-driven methods were particularly sparse. Principal component analysis was used by Velho et al. [38] to derive dietary patterns by aggregating foods into distinct groups. However, principal component analysis and other data-driven dietary patterns approaches are limited by their lack of consideration paid to the health outcomes of interest, such as muscle mass. Hybrid dietary patterns methods, such as reduced rank regression, negate this limitation by combining traditional data-driven dietary pattern approaches whilst considering variation in intermediate response variables, such as nutrients important to muscle health (i.e., protein and omega-3 fatty acids) [51]. Future studies should consider hybrid methods to generate dietary patterns specific to cancer-related muscle loss.

### 4.4. Strengths and Limitations

To the best of our knowledge, this is the first review to summarise the impact of dietary patterns and cancer-related muscle loss. This scoping review has several key strengths. Firstly, rigorous systematic procedures were adhered to in order to generate a robust search strategy and data charting processes, whilst observing the PRISMA protocol extension for scoping reviews [31]. Key gaps in the existing literature have also been identified in an attempt to encourage the prioritisation of important oncological research, such as the development of a muscle-specific dietary index suitable to people with cancer and/or the use of reduce rank regression approaches to derive dietary patterns, which are specific to cancer-related muscle loss, malnutrition, and sarcopenia.

However, these findings should be interpreted in light of some potential methodological limitations. Firstly, specific search terms were explored within three comprehensive research databases to capture all of the relevant studies appropriate to our aim. However, studies may have been unintentionally omitted by the chosen search strategy, such as those which do not explicitly utilise known data-driven or hypothesis-orientated methods to assess dietary patterns (i.e., ketogenic diets). Secondly, consistent with scoping review methodology, a critical appraisal or risk of bias assessment was not conducted on the identified studies and as such the quality of the studies has not been considered. In all, a small number of eligible studies were identified which were heterogeneous in regard to cancer types and treatment modalities, and methods used to assess dietary patterns making it difficult to draw conclusions regarding the most appropriate dietary pattern for people with cancer. However, in line with our aims, we have provided an overview of the existing body of literature regardless of the methodological quality of the studies within.

## 5. Conclusions

This scoping review provided some evidence that a ‘fat and fish’ diet, derived using data-driven dietary patterns methods, may be associated with reduced risk of low muscle mass in people with cancer. Adherence to a priori dietary patterns yielded inconsistent findings. The small number of eligible studies identified and heterogeneity in cancer diagnoses and treatment modalities, and methods used to derive or assess adherence to dietary patterns makes interpretation of the current findings difficult. Further research is needed, employing all dietary patterns approaches, including hybrid methods, and examining malnutrition, and sarcopenia in addition to low muscle mass, to draw a holistic picture of the role of diet in oncological practice. Such research will likely be complementary to our current knowledge of the role of single nutrients and foods on nutritional status and muscle health in cancer and support the development of recommendations for clinical practice.

## Figures and Tables

**Table 1:** Summary of studies investigating the associations between dietary patterns and malnutrition, low muscle mass and sarcopenia in adults with cancer.

Citation and Setting	Study Design and Assessments Timing	Study Population	Cancer Type and Treatment Modality	Dietary Assessment Method	Dietary Pattern Method	Outcomes	Results
Data-driven dietary patterns							
Velho et al. [38] 2019; Portugal.	Design: Prospective (only baseline data reported). Timing: Single assessment at baseline.	*N* = 100 66 men; 34 women Age: 69.5 years	Cancer: Upper and lower gastrointestinal and hepatic-biliary-pancreatic. Treatment: Untreated	Semiquantitative FFQ.	Four PCA derived dietary patterns: (i) high fat dairy products, fried snacks, and processed meat; (ii) legumes, vegetables, and fruit; (iii) fat and fish and; (iv) alcohol, cereal, and animal protein.	Low MM defined as SMI by CT (men: <43 cm^2^/m^2^ if BMI <25 kg/m^2^ or <53 cm^2^/m^2^ if BMI >25 kg/m^2^; women: <41 cm^2^/m^2^).	32% of participants had low MM at baseline. Those in the 2nd and 3rd tertiles of adherence to the ‘fat and fish’ dietary pattern had significantly lower odds of having low muscle mass (OR: 0.30 and 0.24; *p* = 0.02 and *p* = 0.01, respectively).
Hypothesis-orientated dietary patterns							
Baguley et al. [33] 2021; Australia.	Design: RCT (MED diet vs. usual care). Timing: Baseline, 8 weeks, and 12 weeks.	*N* = 23 All men Age: 65.9 years	Cancer: Prostate Treatment: ADT ^1^	One-month diet history.	Mediterranean Diet Adherence Screener.	Total body lean mass by DXA.	MED diet group had significantly lower lean mass at 8-weeks (−1.5 kg; *p* = 0.036) and non-significantly lower lean mass at 12-weeks (−1.4 kg; *p* = 0.06), compared to usual care group. Net differences due to non-significant reductions in lean mass from baseline to 8 weeks which remained until 12-weeks (−1.2 kg; *p* > 0.05) for MED diet group and maintenance of lean mass for the usual care group.
Wang et al. [39] 2021; USA.	Design: Cross-sectional Timing: Single assessment at study enrolment.	*N* = 285 227 men; 58 women Age: 73.8 years	Cancer: Bladder Treatment: TURBT ^2^	FFQ.	Healthy Eating Index 2010.	Low MM defined as SMI (men: ≤52.4 cm^2^/m^2^; women: <38.5 cm^2^/m^2^) by CT.	72% of men and 55% of women had low MM. Diet quality (measured using HEI2010) was not associated with muscle mass (*p* = 0.822).
Dietary patterns assessed using ‘non-traditional’ approaches							
Cohen et al. [34] 2018; USA.	Design: RCT (ketogenic diet vs. American Cancer Society diet). Timing: Baseline and 12 weeks.	*N* = 45 All women Age: 61.5 years (ketogenic diet); 58.6 years (American Cancer Society diet).	Cancer: Ovarian and endometrial. Treatment: Concurrent chemotherapy ^3^	Weekly food records.	Adherence to ketogenic diet assessed by urinary ketone analysis and food records.	Total body lean mass by DXA.	There was no significant difference in lean mass between the ketogenic and American Cancer Society diet groups at 12 weeks (American Cancer Society diet −0.1 kg; Ketogenic diet −0.9 kg, *p* > 0.05 both groups).
Klement et al. [37] 2020; Germany.	Design: Non-randomised trial (ketogenic +/− MAP vs. standard diet). Timing: Baseline (approx. 1 week prior to radiotherapy) and end of radiotherapy (median study duration 35 days). BIA assessed weekly.	*N* = 59 All women Age: 52 years (ketogenic diet); 53 years (standard diet).	Cancer: Breast Treatment: Radiotherapy ^4^	2-day food diaries.	Adherence to ketogenic diet assessed by urinary and blood ketone analysis and food record review.	Total body FFM and derived SKMM by BIA.	Ketogenic diet was associated with a significant, time-independent reduction in FFM and SKMM (−1.23 kg; *p* = 3.4 × 10^−6^ and −0.71; *p* = 1.9 × 10^−4^, respectively). Standard diet was associated with very small, non-significant decrease in FFM and SKMM (>0.005, respectively).
Klement et al. [35] 2021; Germany.	Design: Non-RCT (ketogenic diet +/− MAP vs. standard diet). Timing: Baseline (approx. 1 week prior to radiotherapy) and end of radiotherapy (median study duration 37 days ketogenic diet group; 34 days standard diet group). BIA assessed weekly.	*N* = 41 27 men; 14 women Age: 56 years (ketogenic diet); 65 years (standard diet).	Cancer: Colorectal Treatment: Radiotherapy ^5^	2-day food diaries.	Adherence to ketogenic diet assessed by urinary and blood ketone analysis and food record review.	Total body FFM and derived SKMM by BIA.	Ketogenic diet resulted in significantly reduced SKMM (−0.8 kg; *p* = 0.0032) and non-significantly reduced FFM (−0.2 kg; *p* = 0.205). Standard diet resulted in small non-significant changes in SKMM (−0.2 kg; *p* = 0.520) and FFM (+0.5 kg; *p* = 0.149). Notably, absolute changes in FFM and SKMM correlated with those in TBW and ICW, respectively.
Kammerer et al. [36] 2021; Germany.	Design: Open-label non-randomized nutritional intervention trial (standard diet vs. low carb diet vs. ketogenic diet) Timing: Baseline (Day 2 of 3 week inpatient rehabilitation) and 20 weeks.	*N* = 152 All women Age: 53 years (standard diet); 52 years (low carb diet); 53 (ketogenic diet).	Cancer: Breast Treatment: Neoadjuvant chemotherapy; Tamoxifen; Aromatase inhibitor; Herceptin ^6^	Food diary.	Adherence to each diet assessed via food diary and adherence to ketogenic also assessed by urinary ketone analysis.	SKMM derived from DXA.	Low carb diet resulted in significantly reduced SKMM at 20 weeks (−0.9 kg; *p* = 0.001), whilst ketogenic diet (−0.9 kg; *p* > 0.005) and standard diet (−0.3 kg; *p* > 0.005) resulted in non-significant reductions.

Abbreviations: ADT, androgen deprivation therapy; BIA, bioelectrical impedance analysis; BMI, body mass index; CT, computed tomography; DXA, dual energy X-ray absorptiometry; FFM, fat free mass; FFQ, food frequency questionnaire; MAP, Master Amino Acid Pattern supplement; MED-diet, Mediterranean diet; MM, muscle mass; PCA, principal component analysis; RCT, randomised controlled trial; SMI, skeletal muscle index; SKM, skeletal muscle mass; TURBT, transurethral resection of a bladder tumour; vs, versus. ^1^ Mean time on ADT at baseline = 33.8 months. ^2^ 63.8% underwent TURBT, partial (2.5%) or radical cystectomy (33.6%), with median time since procedure = 2.3 years. ^3^ 28% (ketogenic diet) and 20% (American Cancer Society diet) underwent concurrent chemotherapy. ^4^ 28 fractions of radiotherapy. ^5^ 27 fractions of radiotherapy (ketogenic diet) and 25 fractions (standard diet) +/− chemotherapy. ^6^ Neoadjuvant chemotherapy: 16.1% (standard); 18.5% (low carb); 31% (ketogenic); Tamoxifen: 61.3% (standard); 58.7 (low carb); 34.5% (ketogenic); Aromatase inhibitor: 19.4% (standard); 25% (low carb); 20.7% (ketogenic); Herceptin 19.4% (standard); 14.1 (low carb); 17.2% (ketogenic).

**Table 2:** Summary of dietary patterns from included studies.

Citation	Dietary Pattern	Dietary Pattern Components
Data-driven dietary patterns		
Velho et al. [38]	‘Fat and fish’ dietary pattern	Including olive oil, butter, high-fat snacks (i.e., cookies and chocolates), and fish.
Hypothesis-orientated dietary patterns		
Baguley et al. [33]	Mediterranean Diet Adherence Screener (MEDAS)	A 14-item (yes/no) brief questionnaire, assessing adherence to a Mediterranean-style diet. Higher consumption of olive oil, vegetables, fruits, legumes, fish or shellfish, nuts, meals seasoned with sofrito and preferential consumption of white meat (i.e., chicken, turkey and rabbit), and lower consumption of red and processed meats, butter and cream, sweetened beverages and commercial sweets or pastries indicated better adherence [41]. Notably, question 8 of the MEDAS, regarding alcohol intake, was omitted in this study due to the intervention promoting reduced alcohol intake.
Wang et al. [39]	Healthy Eating Index 2010 (HEI2010)	A 12-component measure of diet quality, assessing adherence to the 2010 Dietary Guidelines for Americans. High consumption of fruits (fruit juice and whole fruits), vegetables, dark green vegetables and beans, whole grains, dairy, total protein foods, seafood and plant proteins and mono- and poly-unsaturated fatty acids, and moderate consumption of refined grains, sodium and empty calories (i.e., energy from solid fats, alcohol and added sugars) indicated better diet quality [42].
Dietary patterns assessed using ‘non-traditional’ approaches		
Cohen et al. [34]	Ketogenic diet	Macronutrient distribution: carbohydrate ~5%, protein 25%, fat 70% of energy intake. Including protein foods (i.e., meat, poultry, eggs and fish), fat-containing foods (i.e., olive and coconut oils, avocado, butter, olives, cheese, cream and a small amount of nuts) and non-starchy vegetables (i.e., salad greens, broccoli and summer squash) and avoidance of all grain and grain products, starchy vegetables and fruits.
Klement et al. [37] 2020	Ketogenic diet +/− MAP	Macronutrient distribution: 75–80% energy from fat, ≤ 50 g carbohydrates per day (≤ 7–10 g per meal). Including whole foods mainly high-quality protein of animal origin (i.e., meat, bone broth, cartilage rich meat and fatty fish), micronutrient dense foods (i.e., vegetables and organ meats), and moderate intake of dairy products (i.e., butter, cheese and fermented products), and avoidance of processed foods, vegetable oils (excluding virgin coconut oils and olive oil), grains and legumes. Fifteen participants received supplementation of 10 g MAP on radiotherapy days [37].
Klement et al. [35] 2021		
Kammerer et al. [36]	‘Low carb’ diet	Macronutrient distribution: carbohydrate 20–30%, protein 20–30%, fat 40–50% of energy intake. Including five portions of fruits and vegetables (i.e., any vegetables, salads and legumes), protein from plant and animal sources (i.e., milk and milk products, low-fat fish, meat and meat products, eggs and nuts) and healthy fat sources (i.e., plant oils such as olive oil, rapeseed oil, coconut oil, linseed oil and hempseed oil), and avoidance of processed carbohydrates and starchy foods (rare full-grain and potato intake).
	Ketogenic diet	Macronutrient distribution: carbohydrate 2–4%, protein 16–18%, fat 80–85% of energy intake. Including fat-containing foods as staple foods (i.e., plant oils, butter, cream, nuts, seeds, olives, avocado, fatty cheese and milk products) and low-sugar fruits and vegetables (i.e., salad and starch-free vegetables) at every meal. Preference given to protein (i.e., eggs, fish, fatty meats, tofu) over carbohydrates.

Abbreviations: MAP, ‘Master Amino Acid Pattern’ (essential amino acids supplement).

## Data Availability

No new data were created or analyzed in this study. Data sharing is not applicable to this article.

## Appendix A

**Table A1:** MEDLINE Complete search strategy.

1	TI cancer* OR AB cancer* OR; TI malignan* OR AB malignan* OR; TI neoplasia* OR AB neoplasia* OR; TI neoplasm* OR AB neoplasm* OR; TI tumor* OR AB tumor* OR; TI tumour* OR AB tumour* OR; MH “neoplasms”
2	TI diet OR AB diet OR; TI “diet* pattern*” OR AB “diet* pattern*” OR; TI “diet* behaviour*” OR AB “diet* behaviour*” OR; TI “diet* quality*” OR AB “diet* quality*” OR; TI “diet* indices” OR AB “diet* indices” OR; TI “diet* index” OR AB “diet* index” OR; TI “diet* score” OR AB “diet* score” OR; TI “eating pattern*” OR AB “eating pattern*” OR; TI “eating habit*” OR AB “eating habit*” OR; TI “Mediterranean Diet” OR AB “Mediterranean Diet” OR; TI “Med-diet” OR AB “Med-diet” OR; TI “Mediterranean-style Diet” OR AB “Mediterranean-stye Diet” OR; TI “Healthy Eating Index” OR AB “Healthy Eating Index” OR; TI “Healthy Diet Indicator” OR AB “Healthy Diet Indicator” OR; TI “Recommended Food Score” OR AB “Recommended Food Score” OR; TI “factor analysis” OR AB “factor analysis” OR; TI “cluster analysis” OR AB “cluster analysis” OR; TI “a priori” OR AB “a priori” OR; TI posteriori OR AB posteriori OR; TI empirical OR AB empirical OR; MH “Diet” OR; MH “Feeding Behaviour” OR; MH “Diet, Mediterranean” OR; MH “Diet, Healthy” OR; MH “Factor Analysis, Statistical” OR; MH “Cluster Analysis” OR; MH “Principal Component Analysis” OR; MH “Empirical Research”
3	TI Sarcopeni* OR AB Sarcopeni*OR; TI “musc* mass” OR AB “musc* mass” OR; TI “lean mass” OR AB “lean mass” OR; TI “lean body mass” OR AB “lean body mass” OR; TI “fat free mass” OR AB “fat free mass” OR; TI myopenia OR AB myopenia OR; TI “musc* wasting” OR AB “musc* wasting” OR; TI “musc* atrophy” OR AB “musc* atrophy” OR; TI “body composition” OR AB “body composition” OR; TI “muscle cross sectional area” OR AB “muscle cross sectional area” OR; TI “musc* strength” OR AB “musc* strength” OR TI “handgrip strength” OR AB “handgrip strength” OR; TI “hand grip strength” OR AB “hand grip strength” OR; TI “grip strength” OR AB “grip strength” OR; TI “musc* weakness” OR AB “musc* weakness” OR; TI “physical function” OR AB “physical function” OR; TI “physical performance” OR AB “physical performance” OR; TI “muscle function” OR AB “muscle function” OR; TI “gait speed” OR AB “gait speed” OR; TI malnutrition OR AB malnutrition OR; TI malnourish* OR AB malnourish* OR; TI undernutrition OR AB undernutrition OR; TI undernourish* OR AB undernourish* OR; MH “Sarcopenia” OR; MH “Muscle Atrophy” OR; MH “Muscle Strength” OR; MH “Muscle Weakness” OR; MH “Body Composition” OR; MH “Physical Functional Performance” OR; MH “Walking Speed” OR; MH “Malnutrition”
4	1 AND 2 AND 3
5	1 AND 2 AND 3 with limiters “English Language” and “Human”

Abbreviations: TI, title; AB, abstract; MH, MESH heading.
